# Supplementary material for: Association of marital and parental status with stress, support, adherence, and quality of life among breast cancer women
Source: PLoS One. 2026 Apr 8;21(4):e0346016. doi: 10.1371/journal.pone.0346016 (PMC13061219; doi:10.1371/journal.pone.0346016)
Supplement: Attachment — Submitted filename: report 1.docx [file pone.0346016.s002.docx]

Introduction

Research Gap Is Not Clearly Stated

The introduction says:

“Although existing data have examined these variables individually…”

…but does not specify:

- What exactly has been studied

- What has not been studied

- Why this study is needed

Suggestion: Explicitly state:

- What previous studies focused on

- What they missed

- Why your study fills an important gap

Missing Key Contextual Variables

The introduction does not mention:

- Cultural norms in Pakistan

- Social expectations of marriage and motherhood

- Why these variables may be particularly important in this setting

Suggestion: Add 1–2 sentences explaining cultural relevance.

Methods:

Inclusion and exclusion criteria are incomplete

You only mention:

- Women aged 18–70

- Confirmed breast cancer

- Exclusion of “life‑threatening comorbidities”

Missing essential details:

- Stage of cancer

- Treatment status (chemotherapy? radiotherapy? surgery?)

- Time since diagnosis

- Metastatic vs. non‑metastatic

- Psychiatric history

- Cognitive ability to complete questionnaires

Data collection method needs more detail

You mention:

“Data were collected through in-person interviews.”

Missing:

- Who conducted the interviews?

- Were they trained?

- Were interviews standardized?

- Were questionnaires self‑administered or interviewer‑administered?

- Language of the survey?

- Were validated translations used?

Instruments section contains repetition and errors

- MSPSS is repeated twice.

- Some sentences are duplicated.

- DASS‑21 description is incomplete and grammatically incorrect.

- MSPSS scoring is misrepresented (the scale is 1–7 per item; your ranges need clarification).

- WHOQOL‑BREF scoring threshold (<60) needs citation and justification

Discussion

Some explanations are speculative and not supported by data

Examples:

- “They don’t have to worry about a husband’s perceptions…”

- “Poor food choices and inactivity due to emotional distress…”

- “Synergistically strengthen emotional resilience…”

These explanations are not measured in your study, so they must be presented as hypotheses, not facts.

Fix: Add phrases like:

- “One possible explanation is…”

- “This may be related to…”

- “Although not assessed in this study…”

Medication adherence interpretation is weak

You state:

“Medication adherence was negatively associated with social support and QoL.”

This is surprising and counterintuitive, but your explanation is incomplete. You mention an Egyptian study but do not finish the sentence.

Fix:

- Complete the explanation.

- Provide possible reasons (e.g., higher symptom burden → more medications → lower QoL).

- Clarify that causality cannot be inferred.
