# Supplementary material for: Association of marital and parental status with stress, support, adherence, and quality of life among breast cancer women
Source: PLoS One. 2026 Apr 8;21(4):e0346016. doi: 10.1371/journal.pone.0346016 (PMC13061219; doi:10.1371/journal.pone.0346016)
Supplement: Attachment — Submitted filename: Response to Reviewers.docx [file pone.0346016.s003.docx]

**Journal Requirements**

**Query 1.** Please ensure that your manuscript meets PLOS ONE's style requirements, including those for file naming. The PLOS ONE style templates can be found at

<https://journals.plos.org/plosone/s/file?id=wjVg/PLOSOne_formatting_sample_main_body.pdf> and [https://journals.plos.org/plosone/s/file?id=ba62/PLOSOne_formatting_sample_title_authors_affiliations.pdf](https://track.editorialmanager.com/CL0/https:%2F%2Fjournals.plos.org%2Fplosone%2Fs%2Ffile%3Fid=ba62%2FPLOSOne_formatting_sample_title_authors_affiliations.pdf/1/010f019b105b2ec8-66533d16-e828-46d5-a8bc-0001d6101540-000000/PEh45vpSPltDUkvAMFRbVN6hd1bjr_rwvQ5OiQ52iHs=241)

**Response:** All the necessary changes have been made in accordance with the PLOS ONE style requirements, including those related to file naming.

**Query 2.** Please provide additional details regarding participant consent. In the ethics statement in the Methods and online submission information, please ensure that you have specified (1) whether consent was informed and (2) what type you obtained (for instance, written or verbal, and if verbal, how it was documented and witnessed). If your study included minors, state whether you obtained consent from parents or guardians. If the need for consent was waived by the ethics committee, please include this information.

**Query 2a.** Thank you for your response regarding the consent type. As previously requested, please specify in the Methods section of the manuscript whether the consent obtained from participants was informed—clearly explaining the nature of the study and emphasizing that participants could withdraw at any time.

**Response:** Thank you for this important clarification. We confirm that informed written consent was obtained from all participants prior to their inclusion in the study. During the consent process, participants were clearly informed about the study’s objectives, procedures, and ethical considerations, and were explicitly assured that their participation was entirely voluntary, with the right to withdraw from the study at any point without any repercussions. The study did not include minors; therefore, parental or guardian consent was not applicable, and no waiver of informed consent was granted. These clarifications have now been explicitly incorporated into the Methods section of the manuscript following page # 6, line # 129-134.

**Query 3.** Thank you for stating the following financial disclosure:

[Deanship of Research and Graduate Studies at King Khalid University for funding this work through the Large Research Project under grant number RGP2/676/46].

Please state what role the funders took in the study.  If the funders had no role, please state: "The funders had no role in study design, data collection and analysis, decision to publish, or preparation of the manuscript."

If this statement is not correct you must amend it as needed.

Please include this amended Role of Funder statement in your cover letter; we will change the online submission form on your behalf.

**Response:** The funder played a role in the manuscript writing, review, and editing, as already stated in the Author Contributions section.

**Query 4.** Thank you for stating the following in the Acknowledgments Section of your manuscript:

[The authors extend their appreciation to the Deanship of Research and Graduate Studies at King Khalid University for funding this work through the Large Research Project under grant number RGP2/676/46]

We note that you have provided funding information that is currently declared in your Funding Statement. However, funding information should not appear in the Acknowledgments section or other areas of your manuscript. We will only publish funding information present in the Funding Statement section of the online submission form.

Please remove any funding-related text from the manuscript and let us know how you would like to update your Funding Statement. Currently, your Funding Statement reads as follows:

[Deanship of Research and Graduate Studies at King Khalid University for funding this work through the Large Research Project under grant number RGP2/676/46]

Please include your amended statements within your cover letter; we will change the online submission form on your behalf.

**Response:** We have removed funding details from the acknowledgment section as per the requirement.

**Query 5.** Thank you for stating the following in your Competing Interests section:

[NO authors have competing interests].

Please complete your Competing Interests on the online submission form to state any Competing Interests. If you have no competing interests, please state "The authors have declared that no competing interests exist.", as detailed online in our guide for authors at <http://journals.plos.org/plosone/s/submit-now>

This information should be included in your cover letter; we will change the online submission form on your behalf.

**Response:** Competing interest section has been updated as per the guidelines.

**Query 6.** When completing the data availability statement of the submission form, you indicated that you will make your data available on acceptance. We strongly recommend all authors decide on a data sharing plan before acceptance, as the process can be lengthy and hold up publication timelines. Please note that, though access restrictions are acceptable now, your entire data will need to be made freely accessible if your manuscript is accepted for publication. This policy applies to all data except where public deposition would breach compliance with the protocol approved by your research ethics board. If you are unable to adhere to our open data policy, please kindly revise your statement to explain your reasoning and we will seek the editor's input on an exemption. Please be assured that, once you have provided your new statement, the assessment of your exemption will not hold up the peer review process.

**Response:** The raw data have been shared in accordance with PLOS ONE guidelines

**Query 7.** Please include a separate caption for each figure in your manuscript.

**Response:** Separate captions for each figure have been included in the manuscript as per the instructions pages 12-13; lines 216 and 239.

Captions of the figures have been included in the manuscript as per the requirements.

**Query 8.** Please include captions for your Supporting Information files at the end of your manuscript, and update any in-text citations to match accordingly. Please see our Supporting Information guidelines for more information: [http://journals.plos.org/plosone/s/supporting-information](http://track.editorialmanager.com/CL0/http:%2F%2Fjournals.plos.org%2Fplosone%2Fs%2Fsupporting-information/1/010f019b105b2ec8-66533d16-e828-46d5-a8bc-0001d6101540-000000/IkZbqHhg-TNT4jjMXndH9wlI-bMJj1Jbk6T1y3fcu8I=241).

**Response:** No supporting information files are included; therefore, no captions or in-text citations are required.

**Query 9:** If the reviewer comments include a recommendation to cite specific previously published works, please review and evaluate these publications to determine whether they are relevant and should be cited. There is no requirement to cite these works unless the editor has indicated otherwise.

**Response:** The reviewer comments did not include any recommendations to cite specific previously published works.

**Reviewer # 1**

**Query # 1. Is the manuscript technically sound, and do the data support the conclusions?**
The study is well-designed and methodologically solid, but some aspects need further clarification or improvement. The research question is relevant and addresses essential gaps in the psychosocial aspects of breast cancer care. The focus on marital and parental status as factors influencing QoL and psychological outcomes is innovative and provides a valuable perspective to existing literature. The sample size is adequate for a cross-sectional study and should allow general conclusions within the study's context. The use of validated instruments is a strength, as it guarantees reliable measurement of the variables.

The manuscript lacks a detailed explanation of how the sample size was determined. Given that this is a cross-sectional study, it would be helpful to include a justification for the sample size, based on power analysis or similar statistical reasoning.

The study doesn’t sufficiently discuss potential confounders, such as socioeconomic status, education, or other personal factors that could influence the results. For example, factors such as income, employment, or healthcare access may also play a significant role in the observed outcomes. If this is not addressed, it must be acknowledged as a limitation of the work. The conclusions are well-supported by the data, but the discussion needs to integrate these findings with existing literature on marital and parental status in breast cancer patients.
Also, many figures were not added and this should be addressed and added.

**Response**: This cross-sectional study included all eligible women with breast cancer who visited the study hospital during the data collection period. A total of 503 participants were included in the analysis, providing statistical power greater than 90% to detect meaningful associations as stated in manuscript page # 5, line # 113-114.

We acknowledge that unmeasured confounders, including socioeconomic status, education, income, employment, and access to healthcare, may have influenced the findings. This limitation has now been added to the manuscript, and future studies are recommended to address these factors for a more comprehensive understanding.

The discussion has been updated to include relevant literature on marital and parental status in breast cancer patients as stated in manuscript page # 13-15 line # 240-287.

**Query # 2. Has the statistical analysis been performed appropriately and rigorously?**
Yes, the statistical analysis appears rigorous, but a few points need to be addressed. The regression analysis should be better described. Authors need to explain which variables were included in the regression model and the rationale behind the selection.

**Response:** The regression analysis has been updated to include a detailed description of the variables in the model and the rationale for their selection as stated in manuscript page # 12-13, line # 218-223.

**Query # 3. Have the authors made all data underlying the findings in their manuscript fully available?**
The manuscript includes data availability statements that align with PLOS ONE’s data-sharing policies.
**Query # 4. Is the manuscript presented in an intelligible fashion and written in standard English?**
The manuscript is well-organized, but several areas could be clarified and improved in grammar and flow. It has a logical structure, making it easy to follow the progression between sections.
Some sentences need more clarity. For example, in the "Results" section, phrases like "psychological discomfort was most pronounced in mothers" could be clearer. Consider using “mothers, particularly those with multiple children, experienced greater psychological distress.” There are some minor grammatical errors and awkward phrasing that should be proofread and fixed.

**Response:** The result section has been updated as per the suggestions as stated in manuscript page # 2, line# 45-46.

The manuscript has been carefully proofread, and the minor grammatical errors and awkward phrasing have been corrected following the page # 4, 5, 6-7, 13, 14, 16, line # 76, 107, 145-146, 219, 224, 229, 232, 236-237, 242, 294-295, 297.

**Query # 5. Limitations That Need to Be Included**

The study's cross-sectional design captures data at a single point in time, limiting the ability to draw causal conclusions or infer long-term trends. Longitudinal studies provide a better understanding of how marital and parental status influence psychological distress, social support, medication adherence, and QoL over time. The study used non-probability sampling from five hospitals in Punjab, Pakistan, possibly causing selection bias and limiting generalizability. Results may not apply to women in rural areas, different cultures, or without healthcare access. The study didn't fully control for confounders like socioeconomic status, education, comorbidities, or treatment type, which could influence psychological outcomes and quality of life. Future research should include these variables to better isolate effects of marital and parental status.
The study's setting in Punjab, Pakistan, may restrict the external validity of the findings. Cultural norms and expectations concerning marriage, family, and caregiving differ significantly across regions. Consequently, the results may not be generalizable to populations in different cultural or geographic contexts. Conducting cross-cultural comparisons would be beneficial in examining how these factors influence women with breast cancer in various settings. Although the study included 503 patients in the final analysis, 110 patients were excluded due to incomplete or missing data. The reasons for missing data are not specified, and the potential impact on the results remains unclear. It should be acknowledged as a limitation.

**Response:** The limitations section has been updated in accordance with the reviewer’s suggestions as stated in manuscript page # 16, line # 289-295.

The handling of missing data has been clarified in the Materials and Methods section, where the exclusion of 110 patients due to incomplete or missing data is now specified following page # 5, line# 114-116.

**Reviewer # 2**

**Query # 1.** In the methods part, exclusion criteria, the authors state the patients with co-morbid life-threatening conditions were excluded. What does this sentence mean? An example on such condition should be provided, moreover, an explanation to why authors made this decision, and how is it going to affect the data generated should also be provided in the main text.

**Response:** The patients with comorbid life-threatening conditions, such as advanced cardiac or renal failure, were excluded to avoid confounding effects on medication adherence, psychological stress, or treatment burden, ensuring that the data reflected breast cancer-specific outcomes as stated in manuscript page # 6, line# 123-126.

**Query # 2.** According to the data released by the World Health Organization, International Agency for Research on Cancer on 24th of FEBRUARY, 2025. ( Globally, most breast cancer cases and deaths occur in individuals aged 50 years and older, who account for 71% of new cases and 79% of deaths ). In this study, the sample included a majority of 181 patients out of 503 that were aged between 25-34, compared to 155 patients aged greater that 45. These numbers contradict the known data regarding the common age of breast cancer, I was hoping to find a part that addresses this issue, even in the discussion section but the authors seemed ignorant of the idea, it was not mentioned as a limitation as well. Such numbers of cases in a young age are strongly linked to family history or genetic mutations like BRCA 1 gene mutation. However, the majority of the responses reported denied having family history of cancer.
This issue is very crucial, and if not properly addressed it may result in an unrepresentative sample and thus a biased study. I kindly ask the authors to investigate why larger numbers of cases in a younger ( unexpected age ) were involved in their study sample. Were all participants from the same geographical area? Are the hospitals included in the study known for treating younger aged patients? What makes these similar\ different from the global population?

**Response:** We appreciate the insightful comment regarding the age distribution in our sample. While global data from the World Health Organization’s International Agency for Research on Cancer indicate that most breast cancer cases and deaths occur in individuals aged 50 years and older, considerable regional variation in breast cancer age distribution exists globally, with distinct patterns evident across Asian populations. Recent studies from the Asia-specific region indicate that a substantial proportion of breast cancer patients present at younger ages compared with Western cohorts, with peak incidence often occurring in the fourth decade of life rather than later decades(1-3).

Although early-onset breast cancer has been associated with family history and genetic mutations such as BRCA1/2, many younger patients do not report a known genetic predisposition. This may partly reflect limited awareness of family medical history and restricted access to genetic testing and medical records in the study setting(4). All participants were recruited from the same geographical region and referral hospitals, which may receive a higher proportion of younger patients with more aggressive disease, potentially leading to overrepresentation of early-onset cases. Lower screening uptake and awareness may also contribute to under-reporting of family history. Notably, the World Health Organization (2024) reports that breast cancer commonly occurs in women without identifiable risk factors such as family history or known genetic mutations(5, 6).

**References:**

1.	Soomro R, Faridi S, Khurshaidi N, Zahid N, Mamshad I. Age and stage of breast cancer in Pakistan: An experience at a tertiary care center. J Pak Med Assoc. 2018;68(11):1682-5.

2.	Zaheer S, Shah N, Maqbool SA, Soomro NM. Estimates of past and future time trends in age-specific breast cancer incidence among women in Karachi, Pakistan: 2004-2025. BMC Public Health. 2019;19(1):1001.

3.	Yuan M, Zhu Y, Ren Y, Chen L, Dai X, Wang Y, et al. Global burden and attributable risk factors of breast cancer in young women: historical trends from 1990 to 2019 and forecasts to 2030 by sociodemographic index regions and countries. J Glob Health. 2024;14:04142.

4.	Zhang Y, Lindström S, Kraft P, Liu Y. Genetic risk, health-associated lifestyle, and risk of early-onset total cancer and breast cancer. J Natl Cancer Inst. 2025;117(1):40-8.

5.	Organization WH. Breast Cancer 2025 [Available from: https://www.who.int/news-room/fact-sheets/detail/breast-cancer.

6.	Ebrahimoghli R, Aghaei MH, Azami-Aghdash S, Houssami N. Uptake of breast cancer screening practices in low- and middle-income countries: a systematic review and meta-analysis. JNCI: Journal of the National Cancer Institute. 2024;117(1):29-39.

**Query # 3.** Line 210-213, if this conclusion is mentioned by other studies in the existing literature, please reference them. Same goes for lines 215-218, 224-225, 229-230.

**Response:** References have been added for all conclusions mentioned as suggested following page # 13-14, line # 243-245, 247-249, 253-254, 258-259.

**Query # 4.** Line 232, it seems like there is a missing part, depression negatively associated with what?

**Response:** The sentence has been clarified to specify that depression was negatively associated with all domains of quality of life as stated in manuscript page # 14, line # 260-262.
**Query # 5.** Lines 248-249 contradict what is mentioned in the Indonesian study mentioned in line 247.

**Response:** The Indonesian study has been replaced with a South Korean study supporting the link between depression and increased medication adherence as stated in manuscript page # 15, line # 278-279.

**Reviewer # 3**

The topic is important, but the research needs some critical corrections.

**Query # 1: Introduction**

Research Gap Is Not Clearly Stated

The introduction says:

“Although existing data have examined these variables individually…”

…but does not specify:

- What exactly has been studied

- What has not been studied

- Why this study is needed

Suggestion: Explicitly state:

- What previous studies focused on

- What they missed

- Why your study fills an important gap

Missing Key Contextual Variables

The introduction does not mention:

- Cultural norms in Pakistan

- Social expectations of marriage and motherhood

- Why these variables may be particularly important in this setting

Suggestion: Add 1-2 sentences explaining cultural relevance.

**Response:** The introductory part of the manuscript has been updated as per the instructions following page # 4-5, line # 86-93, 96-104.

**Query: Methods**

Inclusion and exclusion criteria are incomplete

You only mention:

- Women aged 18-70

- Confirmed breast cancer

- Exclusion of “life‑threatening comorbidities”

Missing essential details:

- Stage of cancer

- Treatment status (chemotherapy? radiotherapy? surgery?)

- Time since diagnosis

- Metastatic vs. non‑metastatic

- Psychiatric history

- Cognitive ability to complete questionnaires

**Response:** The inclusion and exclusion criteria have been updated to incorporate the suggested details, ensuring comprehensive participant selection as stated in manuscript page # 5-6, line # 116-126.

Data collection method needs more detail

You mention:

“Data were collected through in-person interviews.”

Missing:

- Who conducted the interviews?

- Were they trained?

- Were interviews standardized?

- Were questionnaires self‑administered or interviewer‑administered?

- Language of the survey?

- Were validated translations used?

**Response:** The Methods section has been updated to provide additional details on the data collection process as suggested following page # 6, line # 130-135.

Instruments section contains repetition and errors

- MSPSS is repeated twice.

- Some sentences are duplicated.

- DASS‑21 description is incomplete and grammatically incorrect.

- MSPSS scoring is misrepresented (the scale is 1–7 per item; your ranges need clarification).

- WHOQOL‑BREF scoring threshold (<60) needs citation and justification

**Response:** We appreciate the reviewer’s careful reading. Upon review, we confirm that the MSPSS is mentioned only once in the instruments section and all duplicated sentences in the instruments section have been removed to ensure clarity and conciseness. DASS-21, MSPSS, and WHOQOL-BREF tools have been updated as per instructions along with appropriate references as stated in manuscript page # 7-8, line # 143-146, 148-151, 159-162.

**Query: Discussion**

Some explanations are speculative and not supported by data

Examples:

- “They don’t have to worry about a husband’s perceptions…”

- “Poor food choices and inactivity due to emotional distress…”

- “Synergistically strengthen emotional resilience…”

These explanations are not measured in your study, so they must be presented as hypotheses, not facts.

Fix: Add phrases like:

- “One possible explanation is…”

- “This may be related to…”

- “Although not assessed in this study…”

**Response:** We have revised the indications to focus solely on findings directly supported by our study data as stated in manuscript page # 13-14 line # 243-244, 247-249, 253-254.

Medication adherence interpretation is weak

You state:

“Medication adherence was negatively associated with social support and QoL.”

This is surprising and counterintuitive, but your explanation is incomplete. You mention an Egyptian study but do not finish the sentence.

Fix:

- Complete the explanation.

- Provide possible reasons (e.g., higher symptom burden → more medications → lower QoL).

- Clarify that causality cannot be inferred.

**Response:** We have revised the manuscript to provide a complete explanation for the negative association between medication adherence, social support, and QoL, included possible reasons, clarified that causality cannot be inferred, and replaced the Egyptian study with an updated reference following page # 15, line # 266-283.
