# Supplementary material for: Association of marital and parental status with stress, support, adherence, and quality of life among breast cancer women
Source: PLoS One. 2026 Apr 8;21(4):e0346016. doi: 10.1371/journal.pone.0346016 (PMC13061219; doi:10.1371/journal.pone.0346016)
Supplement: Attachment — Submitted filename: Response_to_Reviewers_auresp_2.docx [file pone.0346016.s004.docx]

We want to express our thanks to the Academic Editor for allowing us to revise and improve our manuscript. We truly appreciate the time and consideration you and the reviewers have invested in evaluating our work. The feedback provided is valuable, and we are committed to address all comments carefully to strengthen the quality and clarity of the manuscript. We are grateful for the chance to refine the study in line with the journal’s standards.

**Journal Requirements**

**Query # 1.** If the reviewer comments include a recommendation to cite specific previously published works, please review and evaluate these publications to determine whether they are relevant and should be cited. There is no requirement to cite these works unless the editor has indicated otherwise.

**Response:** No specific previously published works were recommended by the reviewers for citation.

**Query # 2.** Please review your reference list to ensure that it is complete and correct. If you have cited papers that have been retracted, please include the rationale for doing so in the manuscript text, or remove these references and replace them with relevant current references. Any changes to the reference list should be mentioned in the rebuttal letter that accompanies your revised manuscript. If you need to cite a retracted article, indicate the article’s retracted status in the References list and also include a citation and full reference for the retraction notice.

**Response:** The manuscript does not contain any retracted references, so no action regarding such articles is required. The changes made to the reference list have been duly noted and mentioned in the rebuttal letter as per the recommendations.

**Note:** We would like to inform you that the affiliation for one of our authors, **Harris Khurram**, has been updated. Kindly note the revised affiliation in the manuscript:

**Previous:** Department of Science and Humanities, National University of Computer and Emerging Science, Chiniot-Faisalabad Campus, Chiniot, Pakistan

**Revised:** FAST School of Management, National University of Computer and Emerging Science, Chiniot-Faisalabad Campus, Chiniot, Pakistan

We appreciate your attention to this update.

**Query. Review Comments to the Author**

**Query # 4**. Have the authors made all data underlying the findings in their manuscript fully available?

The PLOS Data policy requires authors to make all data underlying the findings described in their manuscript fully available without restriction, with rare exception (please refer to the Data Availability Statement in the manuscript PDF file). The data should be provided as part of the manuscript or its supporting information, or deposited to a public repository. For example, in addition to summary statistics, the data points behind means, medians and variance measures should be available. If there are restrictions on publicly sharing data—e.g. participant privacy or use of data from a third party—those must be specified.

**Response:** Reviewer #1: Yes

Reviewer #2: No

**Response to Reviewer #2 – Query #4:**

Thank you for highlighting the PLOS ONE Data Availability Policy requirements. We fully acknowledge the journal’s policy regarding open and unrestricted access to the data underlying the findings of the manuscript. In compliance with this policy, we have ensured that all relevant underlying data supporting the results of this study are now provided as a Supplementary File.

**Reviewer #1: Minor comments**

**Query:** The sentences in Lines 87-90 should be cited as well as sentences in line 99-102. Line 348 states 'Future longitudinal and cross-cultural studies may further clarify these relationships'. This statement is ambiguous and so there should be a mini section that addresses the future research directions in the area of this research.

**Response:** We have added appropriate citations for the sentences in Lines 87-90 and Lines 99-102. Additionally, we have revised the statement in Line 295 on future research directions in the manuscript following page # 16; line # 295-297.

**Reviewer #2:** Thank you for addressing the issues and doing modifications accordingly.

My major concern was the generalizability of the results of a tertiary hospital data, where cases can differ from global estimates and more aggressive disease form are present. Since the authors declared that the data do not provide generalizable data at the moment and more studies are needed, then the main issue has been addressed and paper is ready for publication.
